# A computational approach to investigating facial attractiveness factors using geometric morphometric analysis and deep learning

**DOI:** 10.1038/s41598-023-47084-x

**Published:** 2023-11-13

**Authors:** Takanori Sano, Hideaki Kawabata

**Affiliations:** 1 Graduate School of Sociology, Keio University, 2-15-45 Mita, Minato-ku, Tokyo, 108-8345 Japan; 2 Faculty of Literature, Keio University, 2-15-45 Mita, Minato-ku, Tokyo, 108-8345 Japan

**Keywords:** Psychology, Human behaviour, Computer science

## Abstract

Numerous studies discuss the features that constitute facial attractiveness. In recent years, computational research has received attention because it can examine facial features without relying on prior research hypotheses. This approach uses many face stimuli and models the relationship between physical facial features and attractiveness using methods such as geometric morphometrics and deep learning. However, studies using each method have been conducted independently and have technical and data-related limitations. It is also difficult to identify the factors of actual attractiveness perception using only computational methods. In this study, we examined morphometric features important for attractiveness perception through geometric morphometrics and impression evaluation. Furthermore, we used deep learning to analyze important facial features comprehensively. The results showed that eye-related areas are essential in determining attractiveness and that different racial groups contribute differently to the impact of shape and skin information on attractiveness. The approach used in this study will contribute toward understanding facial attractiveness features that are universal and diverse, extending psychological findings and engineering applications.

## Introduction

Facial attractiveness plays a vital role in social interactions. For example, facial attractiveness involves positive biases toward mate choice^1^, education^2^, and personality traits^3^. Consequently, numerous studies have been conducted on facial attractiveness factors^4–6^. Experimental studies have shown that the average face obtained by combining multiple face images is rated more attractive than individual face images^7^, and facial symmetry positively influences attractiveness ratings^8^. Furthermore, faces that emphasize sexual dimorphism (masculinity in male faces and femininity in female faces) have higher attractiveness^9^, and sexual dimorphism is related to morphological features formed by sex hormones^4–6^. In female faces, estrogen suppresses bone growth, resulting in rounded cheeks and lips. In male faces, testosterone causes the cheekbones and chin to develop, the brow muscles to rise, the center of the face to protrude forward, and the length of the face from cheek to chin to increase. Thus, facial morphological features, such as facial averageness, symmetry, and sexual dimorphism are important factors in facial attractiveness. In addition to morphological features, facial contrast, luminance, skin condition, smoothness, and blemishes influence one’s perception of facial attractiveness^10–14^. For example, there are gender differences in facial luminance contrast^10^; female faces are deemed more attractive by increasing luminance differences between the eyes/mouth and skin, while male faces are more attractive by decreasing the luminance differences^11^. Thus, many facial attractiveness factors have been identified by experimental methods that investigate facial features based on specific hypotheses.

However, experimental methods cannot consider influences other than hypotheses. Because of the enormous variation in the combination of facial features^15^, it has been difficult to examine facial features comprehensively. In recent years, data-driven approaches that computationally model the relationship between facial features and impression using many face stimuli have been widely used^16,17^. This approach can examine the features that define facial impressions with minimal researcher bias^18^. In recent years, computer graphics (CG) modeling studies^19,20^, geometric morphometrics^21–24^, image statistics^25–27^, and deep learning methods have started being conducted^28,29^.

For example, CG modeling studies showed that faces with large eyes, small noses, and bright skin are attractive^19^, and the attractiveness effects of facial symmetry and averageness do not appear when sexual dimorphism is fixed^20^. In geometric morphometric studies, Nakamura et al.^21^showed that facial features, such as an upward-curving mouth and upturned eyebrows, corresponded to valence. Windhager et al.^22^ suggested that highly attractive and taller men had longer, narrower jaws and wider/fuller lips. Farrera et al.^23^ found that attractiveness is not associated with asymmetry. Furthermore, there is an asymmetric cap (like an asymmetric inverted U-shaped) relationship between body fat percentage and attractiveness^24^. In image statistics studies, Øvervoll et al.^25^ found that images processed with a high spatial frequency filter were preferred for female images, while images processed with a slightly lower spatial frequency filter were preferred for male images. Otaka et al.^26^ showed that skin appearance could be represented by two dimensions: pleasantness and glossiness. Arce‐Lopera et al.^27^ found that color and lightness statistical values correlate with perceived age in skin images. Thus, computational methods have widely revealed the relationship between facial features and impressions.

Furthermore, in recent years, many studies have been conducted using machine learning and deep learning for various engineering applications^30–33^. Particularly, studies on facial attractiveness prediction have attracted much attention^34^. These models achieve highly accurate predictions by learning universal features from many face images. In addition, modeling based on human vision has improved the accuracy of facial attractiveness prediction^35^ and contributed to developing techniques for changing facial image impressions^36^. These methods are also being applied to psychological studies. Facial expression recognition was studied using convolutional neural networks (CNNs) and their hidden layer visualization methods: class activation mapping (CAM)^37^, gradient-weighted CAM (Grad-CAM)^38^, and extremal perturbation^39^. The results showed that an attention map similar to human recognition was extracted^28^. Moreover, sexual dimorphism features are related to facial attractiveness using Grad-CAM^29^.

However, each method had some limitations. CG-generated faces could lack variations in real human faces^40^. Geometric morphometrics requires photographs taken under controlled conditions and the assignment of landmark points. However, these tasks tend to be performed independently within individual laboratories^41^, limiting the images available for research. In addition, it cannot account for information, such as skin, other than morphological features. Image statistics methods design the target image statistics and check the correspondence with facial impressions; thus, there is a limit to designing all possible features. Deep learning methods can consider information other than morphological features, but it is difficult to prepare a large number of control images. For example, the machine learning datasets SCUT-FBP5500^42^ and AffectNet^43^ do not have a uniform face size, position, and background color, which may affect the results^28,29^. Because these methods are often used independently, the relevance of the results from each method is not clear. Furthermore, the correspondence between these computationally obtained results and actual attractiveness perceptions is not detailed.

In this study, we aimed to investigate the essential facial features in attractiveness using a computational approach and clarify the correspondence between these features and actual attractiveness perception. First, we employed geometric morphometrics, the superior method for identifying morphological features, and validated the obtained results with impression evaluation experiments. Using this, we prepared a set of images with uniform size, position, and background. Then, we employed deep learning, the superior method for automatically extracting features inherent in the prepared data.

## Study 1a: analysis using geometric morphometrics

We used the publicly available SCUT-FBP5500^42^ as the dataset, which contains 2,000 Asian male images, 2,000 Asian female images, 750 white male images, and 750 white female images. Each image is labeled with a beauty score, indicating whether the image is attractive. The score is the average of the values evaluated online by 60 volunteers (18–27 years old, average 21.6 years old) using a five-point scale. Because the beauty score of this dataset is described as indicating attractiveness, we treated it as an index of attractiveness in this study. Each face image has 86 landmarks that indicate the morphological position of the face. The landmark points were placed by machine learning and manually corrected by the volunteers to ensure accuracy. In this study, we used images of each race separately for male and female images. Then, we used geometric morphometrics to identify morphological features related to attractiveness. Using this analysis, we manipulated the morphological features associated with attractiveness to create new face images.

### Methods

First, we minimized the distance from the reference by Procrustes analysis^44,45^ and matched the reference positions of the landmark points in the face images. Next, we computed warps from the landmark points using a thin-plate spline^46,47^ and connected and smoothed out the fragments between the landmarks. The warps corresponded to the face morphology variation. We conducted a permutation multivariate regression analysis for each gender, using the warps as the dependent variable and facial attractiveness ratings as the independent variable. That is, we constructed regression models showing the variation of shape information in conjunction with attractiveness ratings. Using the results of the analysis, we visualized and imaged landmark positions. For the imaging, we created formed images for analysis using deep learning methods (see Study 2) and superimposed average images with varying shapes corresponding to attractiveness. We used tpsRegr (version 1.50)^48^ for regression analysis and tpsSuper (version 2.06)^49^ for visualization/imaging.

### Results and discussion

**Figure 1:**
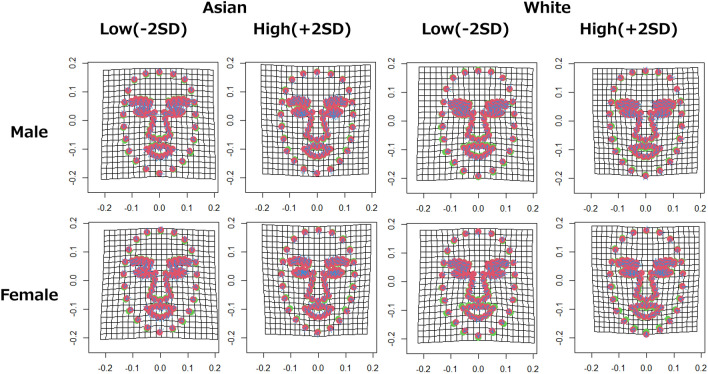
Visualization results of landmark points by geometric morphometrics. The left side is Asian, and the right side is white. The upper row shows the change from the average shape of landmark points corresponding to the attractiveness ratings of male face images. The lower row shows the change from the average shape of landmark points corresponding to the attractiveness ratings of female face images. Red dots indicate landmark points with the average shape, and green dots indicate landmark points with the shape after deformation. The blue arrows indicate the direction of the variation: Low (−2 SD) indicates the result when the attractiveness ratings is manipulated toward −2 SD, and High (+2 SD) indicates the result when the attractiveness ratings is manipulated toward +2 SD.

**Figure 2:**
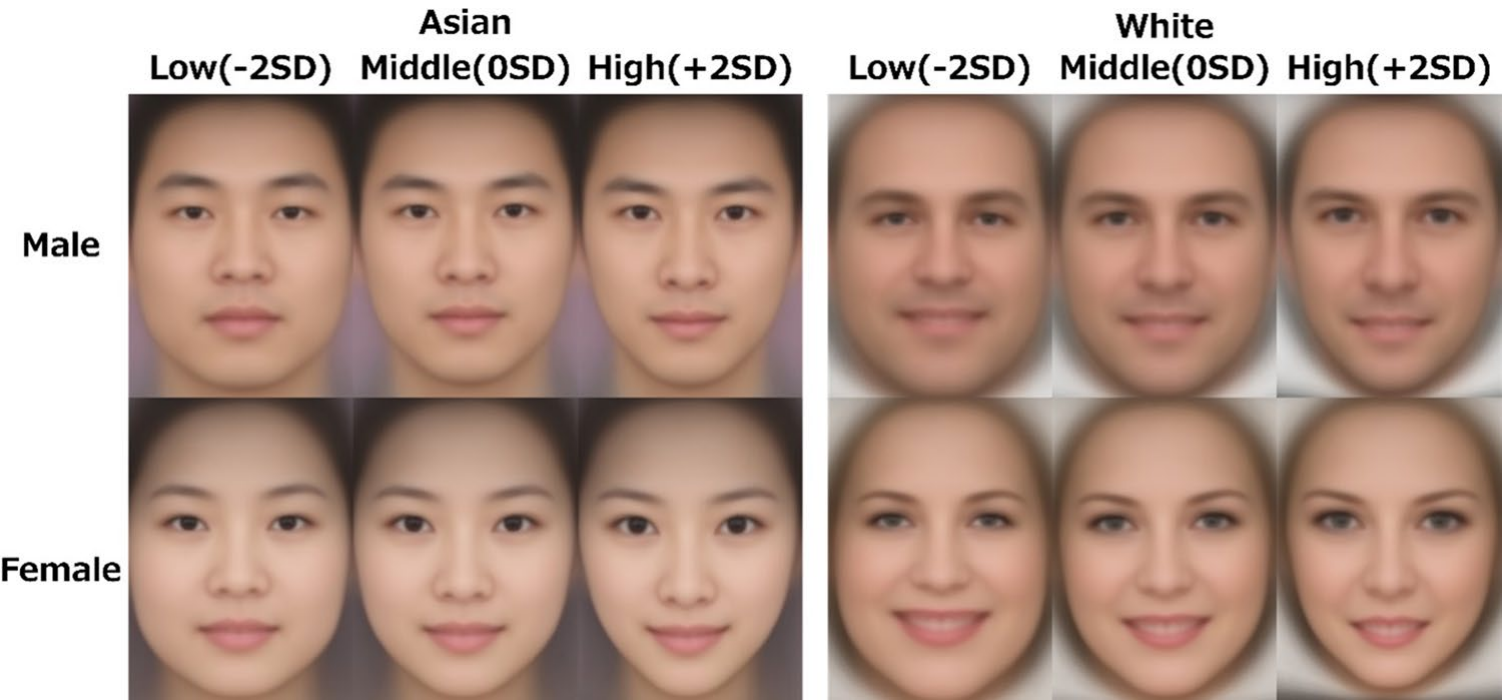
Visualization results of the superimposed average face images using the geometric morphometrics method. The left side is Asian, and the right side is white. The upper panel shows the superimposed average face images corresponding to the attractiveness ratings of the male face images. The lower panel shows the superimposed average face images corresponding to the attractiveness ratings of the female face images. Low (− 2 SD) indicates the result when the attractiveness ratings is manipulated toward − 2 SD, and High (+ 2 SD) indicates the result when the attractiveness ratings is manipulated toward + 2 SD.

The multivariate regression analysis revealed a significant relationship between face shape components and facial attractiveness ratings (Asian male model: explained 3.99% of variance, *p* < 0.001, 1000 permutations; Asian female model: explained 4.42% of variance, *p* < 0.001, 1000 permutations; white male model: explained 3.65% of variance, *p* < 0.001, 1000 permutations; white female model: explained 5.19% of variance, *p* < 0.001, 1000 permutations). One image with missing landmark information was excluded from the analysis. The visualization (Fig. 1) and imaging (Fig. 2) of landmarks using the analysis results showed that attractive faces tended to have large eyes, long and narrow noses, sharp-angled contours, and elevated eyebrows, regardless of gender and race. In addition, the relationship with rising eyebrows tended to be particularly strong in the male images. For the white images, the corners of the mouth appear to be raised, but this may have been influenced by the fact that many analyzed images have smiling faces.

The appearance of large eyes, long and narrow noses, and sharply angular contours as characteristics was consistent with previous studies using computational models^19^. The elevated eyebrows in men may be due to the action of testosterone, which affects facial masculinity^50^.

### Study 1b: Validation test by impression evaluation experiment

In Study 1a, facial features such as eyes, nose, contour, and eyebrows were identified. However, the correspondence between these computationally obtained results and actual attractiveness perception needs to be clarified. Therefore, to verify the correspondence between the results obtained in Study 1a and the perception of attractiveness, we conducted an impression evaluation experiment using images created by geometric morphometry analysis.

#### Participants

Validation tests were conducted independently for Asian and white images. For the validation test of Asian images, the participants were 31 Japanese (14 men, 16 women, one no response; mean age = 23.4 years, SD = 5.51). For the validation test of white images, the participants were 33 Japanese (12 men, 21 women; mean age = 24.4 years, SD = 4.37). A power analysis (α value = 0.05; using the R package SIMR^51^) revealed that these sample sizes yielded at least 80% power respectively. All participants were naive about the purpose of this study. This experiment was approved by the Keio University Ethics Committee in accordance with the Declaration of Helsinki. Informed consent was obtained from the participants.

#### Stimulus

We excluded from the dataset any face images that were clearly celebrities or did not face forward. Then, we selected 25 male and 25 female images from the dataset with attractiveness ratings of − 0.5 SD or less, − 0.5 SD ~  + 0.5 SD, and + 0.5 or more for Asian and white images, respectively. During selection, we employed images in the order of their shape distance from the mean shape calculated by the geometric morphometrics method of Study 1 to increase the change due to shape manipulation. For each of the 75 selected male and female images, we created face images when the shape attractiveness was manipulated to − 2 SD, no deformation (0 SD), and + 2 SD using the model constructed in Study 1a, and prepared 450 face images: 225 for men and 225 for women for Asian and white images, respectively. We clipped all images to an elliptical shape to account for the effect of the background and adjusted their size to 200 × 280 pixels.

#### Procedure

The experimental program was created in PsychoPy^52^ and made executable on a browser by Pavlovia. We sent the uniform resource locator (URL) to participants who agreed with the study content, and they experimented on their personal computers (PCs). In the experiment, participants were asked to rate the facial attractiveness of 450 face images using a five-point scale to match the range of the facial attractiveness ratings in the dataset. Because the dataset includes images collected from the Internet, participants were also asked to rate the familiarity using a five-point scale to check whether celebrities were included in the dataset. Given the burden on the participants, the experiment consisted of two blocks, one to evaluate the male image and the other to evaluate the female image. The order of the blocks was counterbalanced, and the order of image presentation was random. The time to present the face images and answer the questions was unlimited. Participants could take a break at any time, considering fatigue caused by repeated trials.

We used a linear mixed model (LMM) to analyze the obtained face attractiveness ratings as the dependent variable, considering participant and stimulus variability effects. The male image was variable to 0 and the female image to 1. The LMM model was selected by a likelihood ratio test. lmer function of the lme4 package of R was used in the LMM analysis.

### Results and discussion

For the Asian images, first, because none of the obtained results had a mean familiarity score of 3 or higher (M = 1.77, SD = 0.21, min = 1.17, max = 2.39), it was decided to use all the obtained data, judging that celebrities were not included. The model fit well when considering shape change, image gender, and the interaction between the two as fixed effects; participant and stimulus images as random intercept effects; and shape change per participant and per stimulus as random slopes. Therefore, we adopted this model (the full model).

The analysis revealed a shape change effect (Estimate [Est] = 0.12, standard error [SE] = 0.02, t = 8.08, *p* < 0.001); the interaction between shape change and image gender (Est = 0.05, SE = 0.02, t = 2.96, *p* < 0.005) was significant. The image gender effect (Est = 0.16, SE = 0.11, t = 1.56, *p* = 0.12) was not significant. The full model was compared to a reduced model to examine these effects further. The likelihood ratio test results showed a significant difference between the full and reduced model, excluding the effect of shape change (χ^2^ = 47.40, *p* < 0.001). There was also a significant difference between the full and reduced models, excluding the interaction between shape change and image gender (χ^2^ = 8.60, *p* < 0.005).

**Figure 3:**
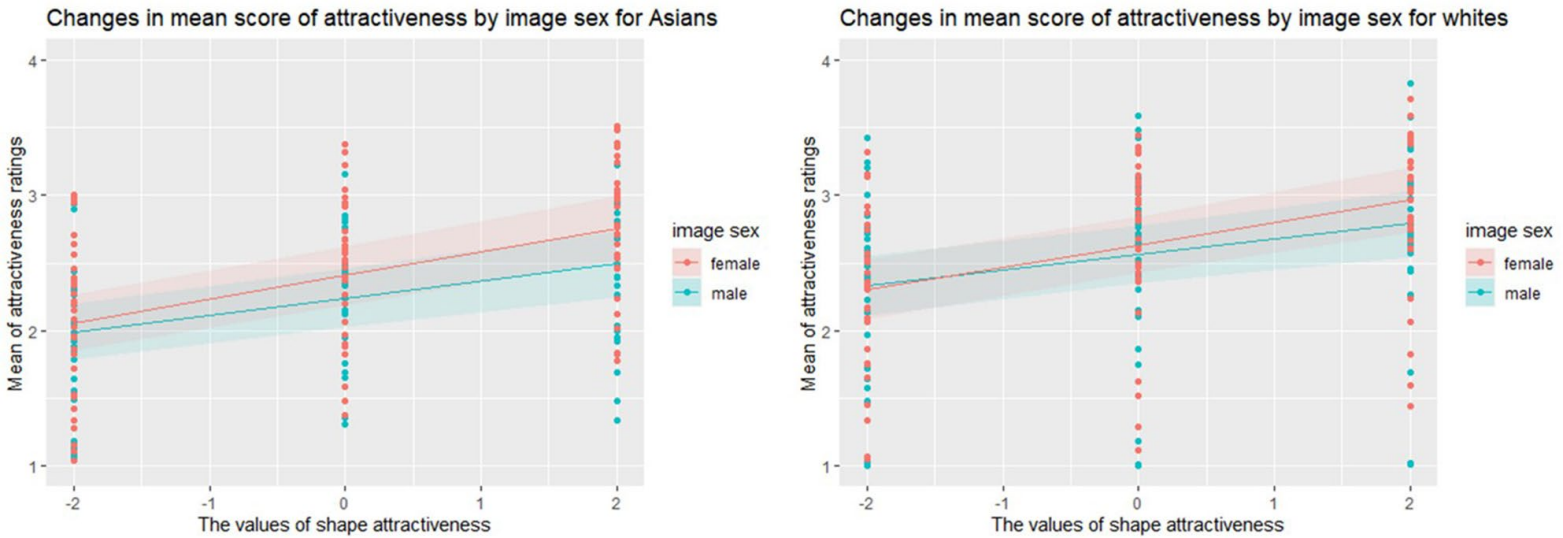
Variation in attractiveness ratings by image gender for Asians and whites. The figure on the left shows data for Asians, and the figure on the right shows data for whites. The values of shape attractiveness show variations of − 2 SD, 0, and + 2 SD. The thick blue line is the regression line of the attractiveness of the male image by linear mixed modeling. The thick red line is the regression line of the attractiveness of the female image by linear mixed modeling. The lightly colored areas represent their respective 95% confidence intervals. The dot value for mean shows the mean value of attractiveness ratings for each participant.

The effect of shape change showed higher attractiveness ratings with shape change in the + SD direction. The effect of the interaction between shape change and image gender showed that the effect of shape change on attractiveness ratings was greater for female than for male images (Fig. 3).

In the results of white images, because none of the obtained results had a mean familiarity score of 3 or higher (M = 1.83, SD = 0.26, min = 1.15, max = 2.61), it was decided to use all the obtained data, judging that celebrities were not included. To make the model the same as the model for Asian images, we employed a model with fixed effects for shape change, image gender, and the interaction between the two, random intercept effects for participant and stimulus images, and random slope for shape change per participant and per stimulus (full model).

The analysis revealed a shape change effect was significant (Est = 0.12, SE = 0.03, t = 4.57, *p* < 0.001). The interaction between shape change and image gender (Est = 0.05, SE = 0.03, t = 1.58, *p* = 0.116); The image gender effect (Est = 0.07, SE = 0.07, t = 1.14, *p* = 0.256) was not significant. The full model was compared to a reduced model to examine these effects further. The likelihood ratio test results showed a significant difference between the full and reduced models, excluding the effect of shape change (χ^2^ = 19.88, *p* < 0.001). There was not a significant difference between the full and reduced models, excluding the interaction between shape change and image gender (χ^2^ = 2.50, *p* = 0.114).

The effect of shape change showed higher attractiveness ratings with shape change in the + SD direction. The slope of shape change was slightly greater for the female images than for the male images, but the effect of the interaction between shape change and image gender not significantly different for the white images (Fig. 3).

LMM analysis revealed significant effects of shape change on attractiveness ratings. This means that the more the shape changes, the higher the actual attractiveness rating. This result suggests that facial features extracted by geometric morphometrics, such as large eyes, long and narrow noses, sharp angular contours, and raised eyebrows, are also critical in actual attractiveness perception. The analysis results also showed that the effect of shape change on attractiveness evaluation was more significant for Asian female than for Asian male images. For the white images, although the result was not statistically significant, the slope was slightly larger for the female images than for the male images. Female faces become attractive when morphological features are changed in a direction that emphasizes femininity, while male faces are not necessarily evaluated as attractive when they are changed in a direction that emphasizes masculinity^9^. Therefore, it is possible that female faces had more consistent features related to attractiveness and that the shape change effect was more significant than for male faces. The LMM analysis suggests that the relationship between facial attractiveness and face shape revealed by the geometric morphometrics corresponds to the actual attractiveness perception, and that the effect is more significant in female images.

## Study 2: analysis using the deep learning model

**Figure 4:**
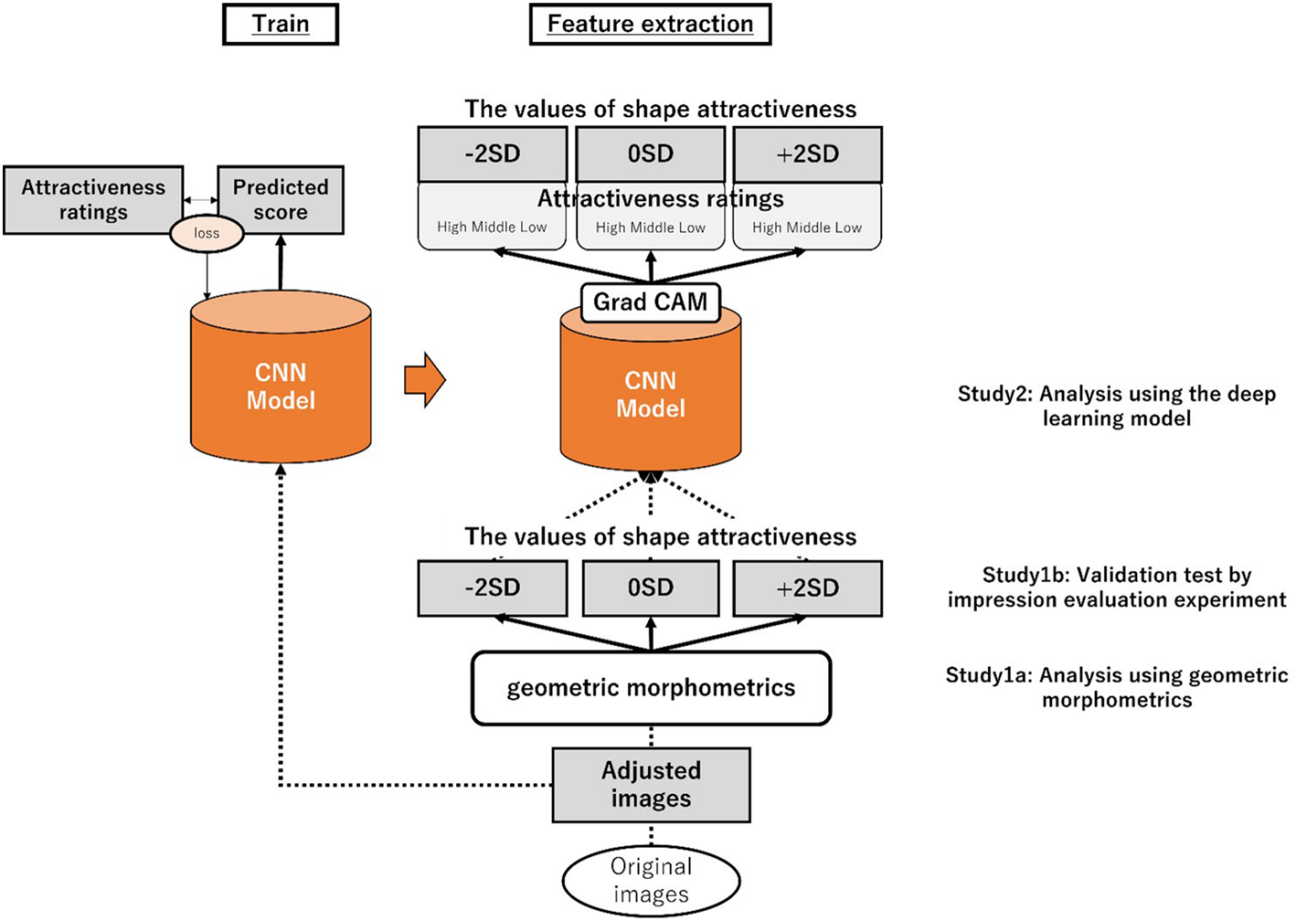
Analysis overview: The convolutional neural network model was trained using the adjusted images created in Study 1a. The constructed model was used to analyze feature extraction. The output results were validated using the face images created in Study1a when the values of shape attractiveness were − 2 SD, 0 SD, and + 2 SD.

We created face images for the training data to prepare the stimuli for analysis of deep learning methods. The training data included the landmark points aligned by the Procrustes analysis used in Study 1. Because several images of the facial orientation of the SCUT-FBP5500 face images were not well controlled, we excluded from the training data the face images in which contours and facial parts were not correctly aligned during the Procrustes analysis. Thus, we prepared 1952 Asian male images, 1786 Asian female images, 584 white male images, and 590 white female images. For accuracy validation, we used the five-division cross-validation method, dividing all data into five parts with four as training data and one as test data, which were then validated for all combinations of divisions. For Asian male images, there were 1562 training data and 390 test data. For Asian female images, there were 1429 training data and 357 test data. For white male images, there were 468 training data and 116 test data. For white female images, there were 472 training data and 118 test data. Then, using these images and the analysis in Study 1, we created face images for each condition, fitting the morphological features in the cases where the attractiveness ratings by shape were high (+ 2 SD), middle (0 SD), and low (− 2 SD). We also clipped each face image to an oval shape to remove the influence of the background. An analysis overview of Study 2 is shown in Fig. 4.

We constructed a CNN model using training data. Subsequently, using the created face images, we extracted features important for predicting facial attractiveness using Grad-CAM. We used tensorflow/keras (version.2.6.0) for the analysis.

### Methods

We tested the prediction accuracy of our model to investigate the structure of a deep learning model. In this study, we treated male and female images as separate datasets to investigate differences in results based on the gender in the image. We performed all tests using a five-part cross-validation method. Based on previous research^29^, we used the Pearson correlation between the label values of the attractiveness ratings of the face images and the predicted results as metrics. For model structure validation, models such as ResNet^53^, DenseNet^54^, and VGG^55^ are expected to achieve high prediction accuracy. However, given the potential impact of complex model structures on visualization results, we employed a simple CNN model in this study. The CNN model has a batch normalization layer^56^ immediately after the CNN layer and a max pooling layer every two layers. We checked the model’s prediction accuracy with two, four, and six CNN layers. The optimization function was Adam, the batch size was 16, the activation function for the output layer was the linear function, the activation functions for the other layers were ReLU, the number of epochs was 500, and the learning rate was fixed at 0.01.

We employed the number of layers that achieved the highest accuracy and built CNN models for each male and female image. Using these models and images created by geometric morphometric analysis, we then confirmed facial features important for attractiveness perception through Grad-CAM, which is excellent at visualizing hidden layers.

### Results and discussion

**Figure 5:**
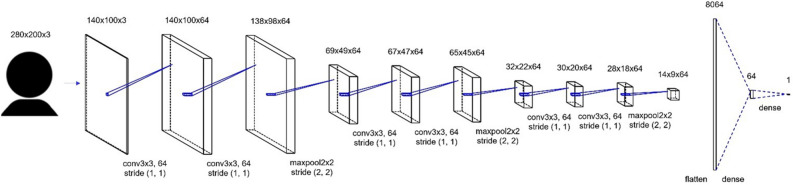
Construction model. The numbers in the upper row indicate each layer’s image and size. The values in the lower row indicate the convolution and max pooling layer values and stride values.

The accuracy of the model structure was verified, and it was found to be highly accurate when the CNN had six layers (Table 1). Therefore, we decided to use a CNN model with six layers. We placed the batch normalization layer immediately after the CNN layer and the Max pooling layer at every two layers of the CNN, as in the validation, and fixed the learning rate at 0.01 with Adam as the optimization function and a batch size of 16. The model diagram is shown in Fig. 5.

**Table 1:** Results of accuracy of five-part cross-validation using Pearson correlation as the criterion.

Model	Race	Sex	Test 1	Test 2	Test 3	Test 4	Test 5	Average
CNN2	Asian	Male	0.79	0.82	0.80	0.81	0.80	0.80
CNN4	Asian	Male	0.84	0.83	0.84	0.82	0.82	0.83
CNN6	Asian	Male	0.85	0.87	0.84	0.84	0.84	0.85
CNN2	Asian	Female	0.83	0.82	0.80	0.83	0.83	0.82
CNN4	Asian	Female	0.82	0.83	0.85	0.84	0.83	0.83
CNN6	Asian	Female	0.84	0.87	0.86	0.82	0.83	0.85
CNN2	White	Male	0.64	0.61	0.62	0.62	0.64	0.62
CNN4	White	Male	0.70	0.61	0.57	0.58	0.67	0.63
CNN6	White	Male	0.65	0.72	0.75	0.71	0.69	0.70
CNN2	White	Female	0.78	0.77	0.79	0.78	0.74	0.77
CNN4	White	Female	0.79	0.80	0.79	0.81	0.73	0.79
CNN6	White	Female	0.84	0.81	0.71	0.80	0.81	0.80

Image sex indicates the gender of the image set used for training and prediction. The model indicates the number of CNN layers used; Tests 1–5 indicate the results of each cross-validation test. The values represent Pearson correlation values, and Average indicates their average values.

Using this model, we also tested the extent of prediction accuracy for cases where training and test data are heterogeneous. The learning epoch was fixed at 100 for each of these cases. The results showed that the prediction accuracy was lower when predicting a person of a different race than when predicting a person of the same race (Table 2). It is possible that there are differences in the features learned by the model depending on the race of the image.

**Table 2:** Results of accuracy for predicting test data for different races using Pearson correlation as the criterion.

Model	Sex	Train data	Test data	Accuracy
CNN6	Male	Asian	White	0.269
CNN6	Male	White	Asian	0.276
CNN6	Female	Asian	White	0.582
CNN6	Famale	White	Asian	0.302

The prediction accuracy when the races of the training and test data are interchanged is shown. A highly accurate model, CNN6, was used. The values represent Pearson correlation values.

**Figure 6:**
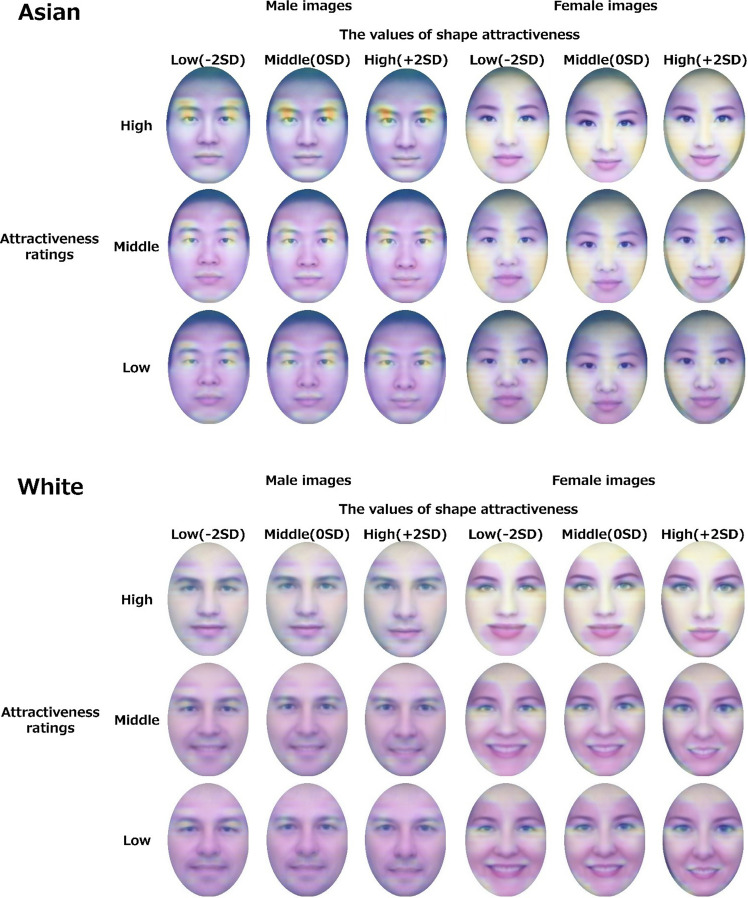
Results of visualization by Grad-CAM. The top figure shows the results for Asian images, and the bottom figure shows the results for white images. The vertical direction shows the attractiveness groups based on the attractiveness ratings originally included in the dataset. The horizontal direction shows the values of shape attractiveness, which is the shape change corresponding to the attractiveness ratings. The left side are male images, and the right side are female images.

Then, we used these models, trained on 500 epochs of aligned same-race source images as training data, to visualize the hidden layer closest to the output layer by Grad-CAM. Because the construction model is a regression model, we treated it as a model with only one class that outputs attractiveness ratings and used the gradient information of the hidden layer for extraction. For the extraction, we used images created by geometric morphometrics. In the case of high (+ 2 SD), middle (0 SD), and low (− 2 SD) shape attractiveness ratings, the top 100 images were selected as the high-, the middle 100 images as the middle-, and the bottom 100 images as the low-attractiveness ratings. These face images were superimposed and averaged for each combination. The stronger the heatmap, the more influential the area for the attractiveness prediction (Fig. 6). For Asian images, the visualization results showed the heatmap was larger around the eyes and eyebrows in the male images. The tendency was larger in the images with high attractiveness ratings. Although the difference in the values of shape attractiveness was not large, the images with high values of shape attractiveness tended to narrow down the active area a little. In addition, the chin area was active. In female images, there was a large heatmap under the eyes and on the forehead in all conditions. Especially in the case of the high-attractiveness ratings, there was a strong tendency for heatmap around the area under the eyes. However, there was little variation in the values of shape attractiveness.

In the white images, the heatmap was more vital across the face for male images when the attractiveness ratings were high. The heatmap was seen around the eyebrows and mouth when the attractive ratings were middle and low. In the female images, there was a strong tendency for heatmap around the area under the eyes, especially when the attractive ratings were high. However, unlike the Asian female images, little heatmap was observed when the attractiveness ratings were middle and low.

The visualization results showed that male images had an overall tendency of heatmap around the eyes, eyebrows, mouth, and chin. The heatmap was greater with higher attractiveness ratings. The results for male eyebrows as an important feature are consistent with previous studies^29^, and the eyebrows and chin may be related to morphological features due to the action of testosterone, which affects facial masculinity^50^. Regarding eye heatmap, the results for eye size are also consistent with the finding that the eyes are an important clue in facial attractiveness^57^. The difference between these activities by the values of shape attractiveness was not large. However, the high values of shape attractiveness tended to narrow the heatmap area slightly, suggesting that morphological features also contribute to attractiveness prediction. However, because the overall fluctuation of heatmap by attractiveness ratings was large, features such as luminance contrast around the eyes and eyebrows may be important predictors of attractiveness using deep learning. Luminance contrast between the eyes/mouth and skin is associated with femininity and masculinity^11^, and sexual dimorphism influences attractiveness perception^9^, suggesting that these features are involved in attractiveness prediction.

The results showed greater heatmap around the eyes, especially in the female images with high attractiveness ratings. The heatmap around the eyes in the female images is consistent with a previous study^29^. The results may also be related to the influence of luminance contrast^10^ and the psychological finding that women are more attractive by increasing the luminance contrast between eyes/mouth and skin^11^.

While the eye area and the luminance contract were common elements in Asian and white images, there were racial differences in the case of high attractiveness ratings in male images and in the case of middle and low attractiveness ratings in female images. When the model's accuracy was tested for different races, the prediction accuracy was lower than that for the same race, suggesting that the model may have learned different characteristics depending on race.

## General discussion

Study 1a showed that large eyes, elongated noses, sharp-angled contours, and raised eyebrows are important features for attractiveness regardless of gender. The eyebrows were particularly pronounced in the male images. Study 1b showed that the relationship between facial shape and attractiveness revealed by geometric morphometrics corresponds to actual attractiveness perception. The effect was more significant for Asian female than for Asian male images. Study 2, using Grad-CAM, showed that male images were active around the eyes, eyebrows, and chin with higher attractiveness ratings resulting in greater heatmap. In addition, the higher the values of shape attractiveness, the slightly narrower the heatmap area. The female images showed heatmap around the area under the eyes in all conditions with greater heatmap for higher attractiveness ratings. Consistent with Studies 1 and 2, eyebrows and eyes in male images were identified. In Study 1, elevated eyebrows were associated with attractiveness. In Study 2, the higher the attractiveness ratings, the more active the eyebrow area, and the higher the values for shape attractiveness, the more the area was narrowed.

In the findings of this study about the eyebrows of male faces, previous studies using geometric morphometrics showed that eyebrow thickness was related to the perception of masculinity^58^. In a previous deep learning study, Grad-CAM showed that heatmap corresponds to the brow region's attractiveness in male images^29^. Furthermore, protruding eyebrows are associated with facial masculinity^4–6,50^, and faces with enhanced sexual dimorphism are more attractive^9^. The results of this study are consistent with those of previous studies. CG modeling studies^19^ and various psychological studies^57,58^ show eyes to be an essential feature for attractiveness. In Study 1b, the effect of shape change, which encompassed changes in eye size, nose width and length, contour sharpness, and eyebrow angle, was also significant. Therefore, the association between male eyebrows and eyes and attractiveness can be an essential factor both as an image feature and as perceived by observers.

Study 1 showed a relationship between eye size and attractiveness in female images. The effect of eye size can be explained by the fact that eyes significantly influence the overall attractiveness rating of the face^59^. Study 2 showed heatmap near the lower part of the eye in female images. This heatmap may be related to the effect of luminance contrast between the eyes and skin on attractiveness^10^. In Study 1b, the effect of shape change on attractiveness ratings was more significant for Asian female than for Asian male images. For the white images, although the result was not statistically significant, the slope was slightly larger for the female images than for the male images. These results suggest that areas related to the eyes are important for a woman's facial attractiveness. However, Study 2 differed from Study 1a in that under the eye, rather than the eyes themselves, was the critical feature in predicting attractiveness. Moreover, although the effect on shape change in Asian female images was greater in Study 1b than in Asian male images, the heatmap was mainly in the skin region rather than in the morphological features in Study 2. These results indicate that the features that are important factors vary depending on the method employed, representing the complexity of facial attractiveness components. In fact, there are complex relationships, such as the illusory eye size effect of makeup on the skin area^60,61^, but the details require further study.

In Study 2, when the attractiveness rating was high, the heatmap focused more on parts such as eyes and eyebrows in Asian male images. In contrast, the heatmap was more spread over the entire image in white male images. When the attractiveness ratings were middle and low, the heatmap under the eyes was more comprehensive for Asian female images, while the area was narrower for white female images. It is suggested that the facial features learned by the deep learning model differ depending on race. In the case of Asian male images, facial parts were more important than skin information. For male attractiveness, shape information was reported to be more important than reflectance information^62^, whereas another study emphasized the importance of the attractiveness of color information^63^. A computational study of the contribution of shape and reflectance to East Asian facial attractiveness suggested that shape information was more important^19^. This is consistent with the finding in this study that factors related to shape are more important for Asian male faces.

For female images, differences were observed in the size of the skin area in the heatmap. The dataset used in this study included many women's faces with makeup, which may have had an effect. In Asian cultures, being fair-skinned is an important component of female beauty^64^, and this difference in values and makeup practices may have influenced the extent of the heatmaps. Although further verification of the details is needed, these results suggest that racial differences that could not be observed using morphological analysis alone may be extracted using deep learning methods.

The face stimuli created in Study 1 changed shape from − 2 SD to + 2 SD—the range of scores present in the image data set—and the results showed a good fit of the LMM. This result suggests that a linear relationship exists between observers’ perception of attractiveness and changes in facial morphological features such as eye size, nose width and length, contour sharpness, and eyebrow angle. However, Windhager et al.^24^ showed an asymmetric cap (like asymmetric inverted U-shaped) relationship and attractiveness with shape changes ranging from − 5 SD to + 5 SD. Because it has been repeatedly reported that averageness is an important factor in facial attractiveness^4–7^, it can be inferred that significant deviations from the average may have a negative effect. Therefore, subsequent studies on the nonlinear relationship between shape change and attractiveness perception over a wide range are expected.

In previous studies, geometric morphometrics and deep learning methods have been conducted independently, and thus the concluding results have depended on the analysis method. By taking the approach proposed in this study, the results can be captured more multifacetedly. In fact, the results suggest that the eyes and eye area are important in attractiveness, while detailed features such as eyebrows, contours, and overall skin information are obtained differently depending on the analysis method. That is, this approach is superior in that it can examine universal features that are independent of the method. In addition, this study adopted the process of validation testing by impression evaluation experiments to check the correspondence between the computationally calculated results and the actual attractiveness perception. Such an approach will lead to a more detailed understanding of psychological knowledge because it enables the evaluation of cognitive responses to extracted facial features that are important in a data-driven manner.

There are several limitations to this study. First, we have yet to be able to compare results with models with high prediction accuracies, such as ResNet, DenseNet, and VGG, or with relatively new visualization methods, such as Grad CAM++ ^65^ and Score CAM^66^. Second, a Procrustes analysis was performed to control the size and position of many face images. However, the attractiveness ratings were analyzed using the scores before position adjustment. Although there is a limit to the availability of a dataset with all the face image shooting conditions, number of images, and impression scores, further research that considers these factors is expected in the future. Third, the nationalities of the raters in the dataset used in this study were unknown, but the participants in the validation experiment in Study 3 were Japanese. In addition, since the ages of the raters in the dataset ranged from 18 to 27 years old, relatively young participants were collected in Study 3. While facial attractiveness is highly consistent within and across cultures^67^, there are also cross-cultural differences in facial color preferences^68^. Similarly, attractiveness ratings may change depending on the age of the raters^69^. Therefore, future research on the characteristics of individual raters is expected. Fourth, although there were variations in the facial expressions or age of the images used in this study, the model was constructed without separating the model by facial expression or age. While this has the advantage of keeping the number of training data since all data can be used, these various uncontrolled factors may have influenced the results. For example, smiling people give a more attractive and positive impression than non-smiling people^70,71^, so we cannot isolate the possibility that facial expressions influenced the results. Furthermore, it has been reported that attractiveness decreases with age^69^. This effect must also be considered. To enable these analyses, it is expected that large datasets with labels for facial expressions and age, in addition to attractiveness and facial landmarks, will be available in the future. Finally, although the possibility of a contribution from skin regions was identified in this study from Study 2, further experiments are expected to be conducted to calculate more detailed image features and to verify their effects on the contribution of morphological features and texture information.

## Conclusion

This study investigated the important facial features in facial attractiveness using geometric morphometrics and deep learning methods. The results showed that large eyes, a long and narrow nose, sharp angular contours, and raised eyebrows were associated with geometric morphometrics. The deep learning method was associated with eyebrows and eyes for male images and the area under the eyes for female images. Furthermore, impression evaluation experiments showed that these features correspond to the actual perception of facial attractiveness. Overall, features related to the eyes and eye area were extracted, suggesting that these features are essential in facial attractiveness. In addition, it was suggested that differences in the contribution of shape and skin information to attractiveness between Asians and whites could be extracted using this approach.

Our approach contributes to understanding highly universal features in facial attractiveness and extends psychological knowledge. It is also expected that combining research on generative modeling based on perceptual mechanisms will contribute to further understanding of attractiveness factors and various engineering applications^34^ such as face editing^33^ and face beautification^32^. Therefore, it is crucial to continue research based on this approach with an eye toward both psychological and engineering perspectives.

## Data Availability

The created face image data, attractiveness and landmark point data, and R code are available at https://osf.io/b8taz/.
